# Analysis of COVID-19 Vaccine Adverse Drug Reactions Reported Among Sultan Qaboos University Hospital Staff

**DOI:** 10.18295/squmj.3.2024.026

**Published:** 2024-05-27

**Authors:** Bushra H. Al Busaidi, Intisar M. Al Riyami, Hashim Ba Wazir, Ibrahim S. Al Zakwani

**Affiliations:** 1 Department of Pharmacy, Sultan Qaboos University Hospital, Sultan Qaboos University, Muscat, Oman; 2 Department of Medicine, Sultan Qaboos University Hospital, Sultan Qaboos University, Muscat, Oman; 3 Department of Pharmacology & Clinical Pharmacy, College of Medicine and Health Sciences, Sultan Qaboos University, Muscat, Oman

**Keywords:** Pfizer-BioNTech (BNT162b2), Oxford-AstraZeneca (ChAdOx1 nCoV-19), COVID-19 vaccines, COVID-19, Adverse Drug Reactions, Oman

## Abstract

**Objectives:**

This study aimed to report any suspected adverse drug reactions (ADRs) experienced by all vaccinated staff and students in a tertiary teaching hospital following COVID-19 vaccination.

**Methods:**

This retrospective study was conducted during the COVID-19 vaccination campaign at Sultan Qaboos University and Hospital in Muscat, Oman, from August to September 2021. An online survey was generated and sent to all staff and students via email and text messages. An announcement was made on the hospital website with a link to the survey.

**Results:**

A total of 8,421 individuals reported being vaccinated at least once with a total of 11,468 doses administered from January to July 2021; 8,014 staff and students received the Pfizer-Biotech vaccine while 3,454 staff and students received the Oxford-AstraZeneca vaccine. The survey received a total of 3,275 responses (response rate = 38.8%). Of these, 741 individuals (22.6%) experienced an ADR after vaccination and 67.2% (n = 498) were females (*P* <0.001). The majority of the ADRs reported were fever and chills (19.7%) followed by localised pain and swelling at the injection site (18.8%). Other ADRs such as hair loss (0.5%) were reported, and one staff/student reported a clot in the right leg. Among the responders, 27.0% considered their ADRs as mild while 25.0% considered them as severe.

**Conclusions:**

In the study cohort, mild symptoms of COVID-19 vaccines were reported. Females experienced more ADRs compared to males. Long-term observation of ADRs to the vaccines and follow-up monitoring should be done on subjects to preclude any unwanted effects.

**Advances in Knowledge**

- *This study found adverse drug reactions (ADRs) to COVID-19 vaccines in a long-term follow-up, and some of these ADRs have not been previously documented in the literature.*

**Applications to Patient Care**

- *Healthcare providers should be aware of other unreported ADRs and be vigilant in monitoring patients while administering COVID-19 vaccines.*

Two COVID-19 vaccines were available in Oman—the Pfizer-BioNTech (BNT162b2) and the Oxford-AstraZeneca (ChAdOx1 nCoV-19). Both received emergency use authorisation from the Food & Drug Administration (FDA) of the USA and the Medicines and Healthcare Products Regulatory Agency (MHRA) of the UK as they have shown acceptable efficacy and safety profile in patients in the first and second phases of the clinical trial.^1^–^5^ Given that the vaccine is relatively new, there were no long-term adverse drug reactions (ADRs) reported or studied. In clinical trials, the most common ADRs reported for both vaccines were injection site pain, headache and fatigue.^1^–^3^ On the other hand, some serious ADRs have been observed for both vaccines.^6^ In the Oxford-AstraZeneca (ChAdOx1 nCoV-19) vaccine trial, 0.7% of serious ADRs were reported in the vaccine group, while in the Pfizer-BioNTech (BNT162b2) vaccine trial, 0.6% of serious ADRs have been reported.^6^^,^^7^

COVID-19 vaccination campaigns were held across the globe to ensure proper eradication of the virus. Sultan Qaboos University Hospital (SQUH) in Muscat, Oman, started the vaccination process initially to vaccinate healthcare providers, who have direct contact with admitted patients infected with the COVID-19 virus. Eventually, the vaccination campaign at SQUH was extended all hospital staff, followed by the university staff and students.

This study aimed to evaluate ADRs outside the context of clinical trials and provide more context on the long-term possible ADRs at SQU.

## Methods

This observational retrospective study was conducted after the COVID-19 vaccination campaign from August to September 2021, which took place at SQU and SQUH in Muscat, Oman. All staff, including students, were scheduled for vaccination. The dates were announced ahead of the campaign start date. All individuals were asked to complete a form with information such as age, contact number and any known allergies requested by the infection control team. Following ethics approval, a list of all vaccinated individuals with their details was provided to the infection control team. The study included all individuals older than 12 years who received the first or second dose of the vaccine at SQUH.

Using an online Google form (Google LLC, Mountain View, California, USA), a short survey was generated in Arabic and English. This consisted of 14 questions that were easy and fast to complete. It took approximately 2 minutes or less to complete the survey. Questions were mainly related to ADRs experienced after the vaccination either after the first dose, second dose or both doses. There were also questions related to the recovery from the ADR, outcomes, as well as the effect of the ADR on going back to work.

The survey was sent via the university email to all staff and students. Moreover, it was announced on the hospital website that a QR scan code and link to the survey were also accessible. There was also an initiative for sending free text messages by Omantel (Oman Telecommunication Company, Muscat, Oman) to all vaccinated staff with a direct link to the survey. Much emphasis was placed by all pharmacists in sending the survey link through different clinical groups and reminding healthcare professionals to complete the survey. The survey was voluntary and not compulsory.

Descriptive statistics were used to describe the data. For categorical variables, frequencies and percentages were reported. Differences between groups were analysed using Pearson’s χ^2^ tests (or Fisher’s exact tests for cells <5). For continuous variables, mean and standard deviation were used to present the data. An a *priori* two-tailed level of significance was set at the 0.05 level. Statistical analyses were conducted using STATA, (STATA Corporation, College Station, TX, USA), Version 16.1.

The Medical Research Ethics Committee of the College of Medicine and Health Sciences at SQU approved this study (MREC #2499).

## Results

Between January to July 2021, a total of 11,468 doses of COVID-19 vaccines were administered corresponding to 8,421 individuals (>12 years old); a total of 8,014 individuals received the Pfizer-BioNTech vaccine, of which 80.0% (n = 6,414) received only the first dose and 20.0% (n = 1,600) participants also received the second dose. A total of 3,454 individuals received the Oxford-AstraZeneca vaccine, of which 55.2% (n = 1,909) received the first dose while 15.7% (n = 1,545) received the second dose.

Among the 8,421 subjects who were vaccinated, only 38.9% (n = 3,275) responded to the survey distributed in which significantly more females responded than males (57.0% versus 43.0%). The majority of responses were filled by adults whereas 49.0% were by participants aged 12–30 years followed by 29.0% aged 31–40 years. Only 19.0% of the responses were from the age group 41–50 years while the elderly contributed to only 1.0% of the responses. Among all responses, 22.6% (n = 741) experienced an ADR. Among the participants who responded, 65.0% received the Pfizer-BioNTech vaccine and 38.0% received the Oxford-AstraZeneca.

A total of 39.0% of individuals who completed the survey were healthcare providers who worked at the hospital, 19.0% were students and 9.0% were university staff. A total of 35.0% of individuals were categorised as ‘others’.

**Figure 1:**
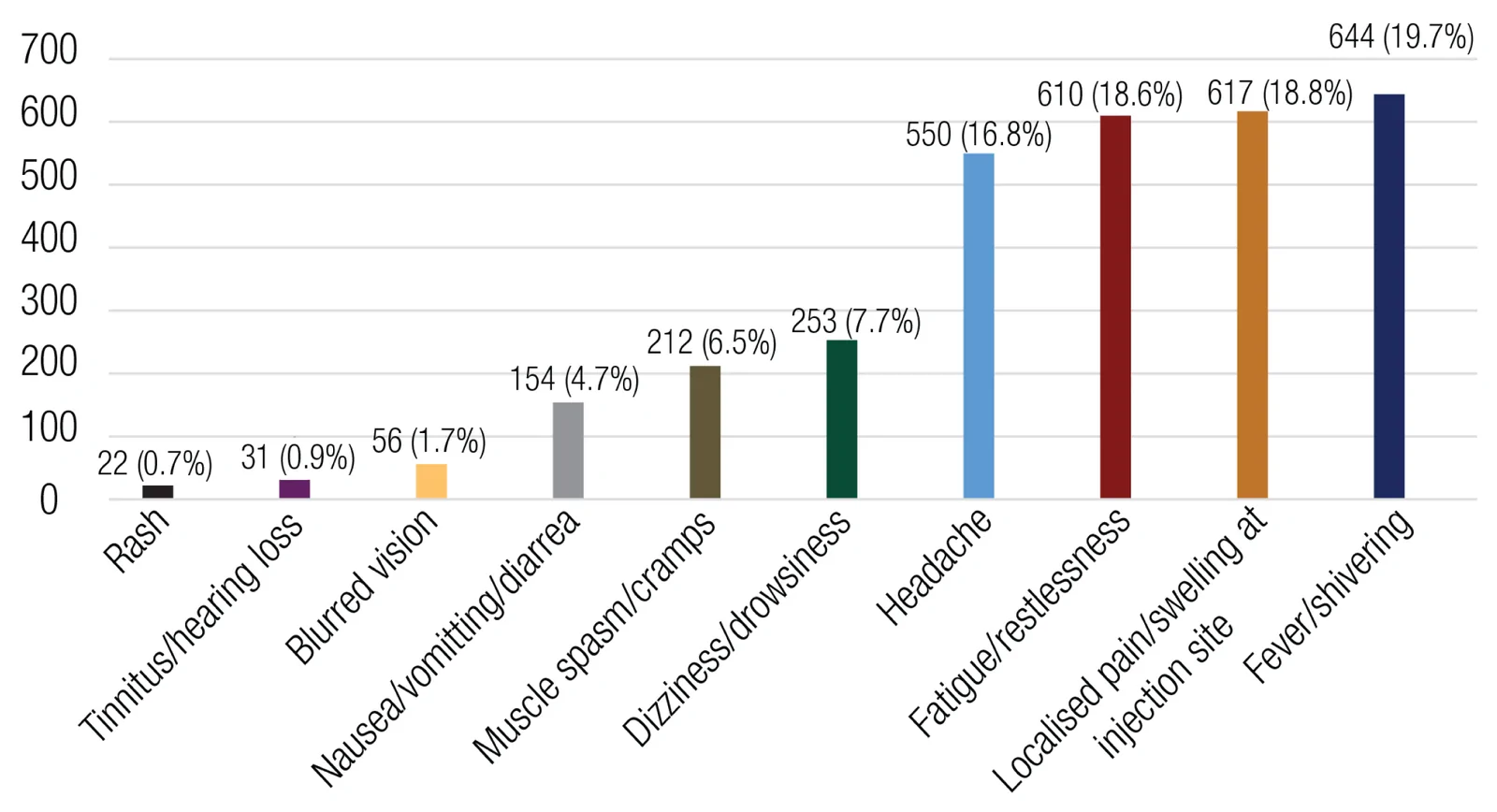
Adverse drug reactions experienced by responders to COVID-19 vaccinations (n = 3,275).

**Figure 2:**
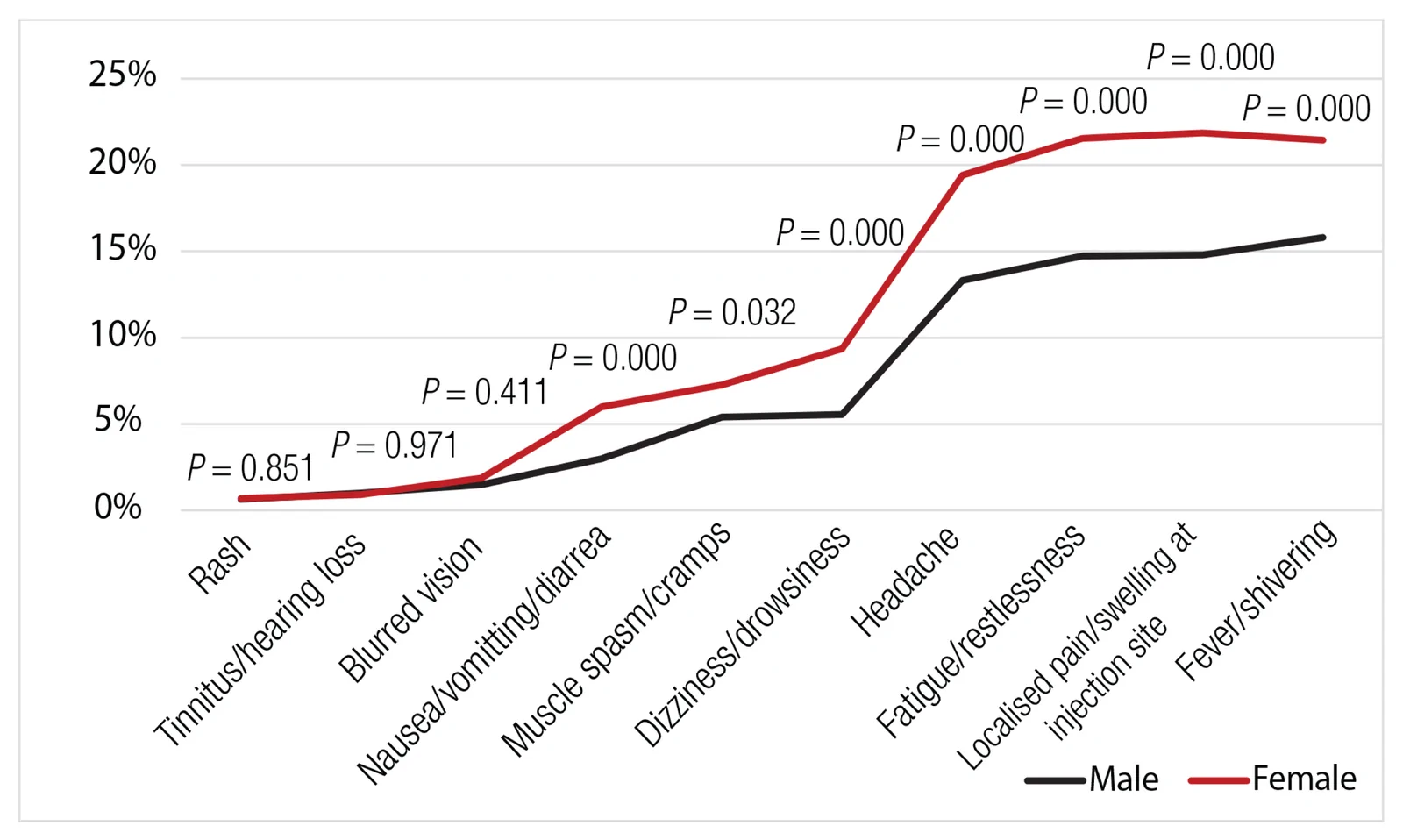
Gender distribution among those who experienced adverse drug reactions to COVID-19 vaccinations.

The reported adverse effects were remarkably similar. An average of 14.5% of all reports were fever and shivering, localised pain at the injection site, fatigue, restlessness and headaches. This is followed by dizziness (7.7%) and muscle cramps (6.5%). There were 31 (1.0%) individuals who experienced tinnitus and hearing loss [Figure 1]. The results showed a significant increase in ADR incidents in females compared to males (*P* <0.001) [Figure 2].

**Figure 3:**
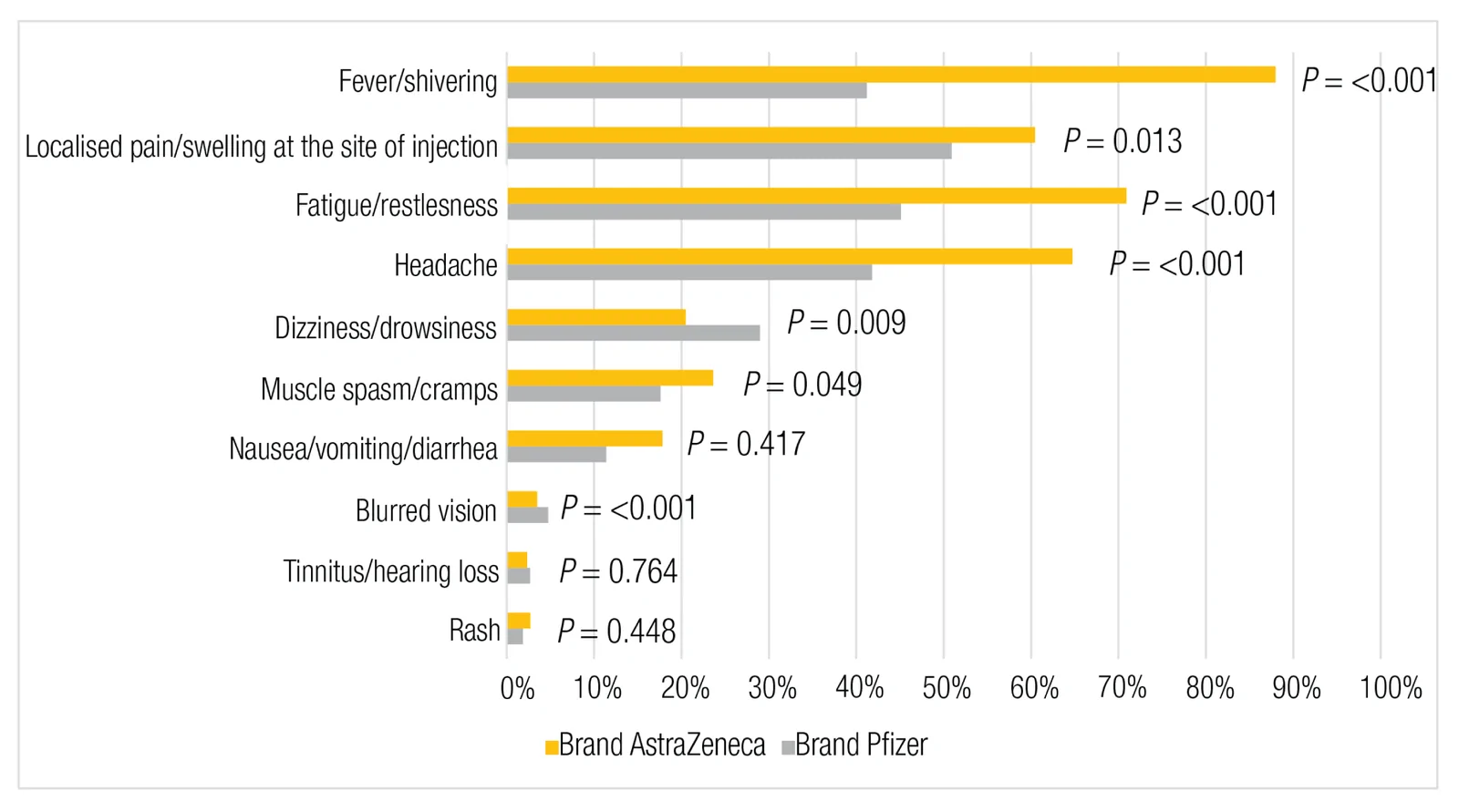
Vaccine brand distribution among those who experienced adverse drug reactions.

Among the 2 vaccine brands, there were significant differences in ADR distribution among males and females. ADRs, such as fever and shivering (*P* <0.001), localised pain and swelling (*P* = 0.013), fatigue and restlessness (*P* <0.001) and headache (*P* <0.001), were also significantly more prevalent in the Oxford-AstraZeneca vaccine compared to the Pfizer-BioNTech vaccine [Figure 3].

Other ADRs were reported but were not listed in the distributed survey. There were 61 reports of body pain, which included muscular and bone pain. Other reports included chest tightness (n = 15), irregular menstrual cycle (n = 12), flu-like symptoms (n = 15), swollen lymph nodes (n = 5), loss of appetite (n = 7), palpitations (n = 5), loss of smell (n = 5), hypotension (n = 5), insomnia (n = 4), hair loss (n = 4) and neuropathic pain on the fingertips (n = 3).

## Discussion

There are limited data regarding the long-term side effects of the COVID-19 vaccines. This is due to the emergency-use authorisation by both the MHRA and the FDA, and due to them being released only approximately 2 years ago. In this retrospective study on the SQUH COVID-19 vaccination campaign, the rate of adverse effects from 2 types of COVID-19 vaccines—the Pfizer-BioNTech and the Oxford-AstraZeneca—were investigated. Currently, only scant reports of long-term side effects are available and the number of participants enrolled in these clinical trials was very low.^8^

The majority of participants who experienced an ADR received the Pfizer-BioNtech vaccine (65.0%), which was due to the abundant availability of this type of vaccine initially at SQUH. Most of the responders in this study were females (57.0%), who also reported a higher incidence of adverse events (67.0%) compared to males. This is in line with two other published reports by Dutta *et al*. and David *et al*. where they reported higher adverse effects in females compared to males.^9^^,^^10^

In this cohort, there was no difference in age distribution among persons who have experienced an ADR. However, this may be due to the small number of participants aged >50 years (6.0%) while the majority of the participants were aged 12–30 years (46.0%). In a study of a cohort that included all age categories, David *et al*. did not observe any age difference in the development of ADRs between younger participants compared to the elderly (80 years and older).^10^ Higashino *et al*. observed that vaccine recipients aged 30–69 years experienced significantly more ADRs compared to those aged 18–29 years.^11^

In the current study, ADRs reported were mostly fever and shivering (19.7%), localised pain and swelling (18.8%), fatigue and restlessness (18.6%) and headache (16.8%). These 4 most common ADRs were more pronounced in individuals who received the Oxford-AstraZeneca vaccine than those who received the Pfizer-BioNtech vaccine. However, dizziness and drowsiness were experienced by 7.7% of individuals and it was mostly by participants who received the Pfizer-BioNtech vaccine compared to the Oxford-AstraZeneca vaccine.

There were no serious adverse effects reported in the current cohort, such as pulmonary embolism, myocarditis, thrombosis or stroke, unlike the incidents reported by Klein *et al*.^12^ This could be due to either underreporting, small sample size or due to the incidence not occurring in the first place.

In the literature, thrombotic events were documented concerning the Oxford-AstraZeneca vaccine more than other vaccines, in which some cases were fatal.^8^^,^^13^ A case study published by SQUH reported an extensive deep vein thrombosis and pulmonary thromboembolism in a 59-year-old patient who received the Pfizer-BioNTech vaccine.^14^ The occurrence of thrombosis was not proven to have a direct association with the vaccines. However, further studies are warranted to corroborate this association.

In the Arab population, as described by Hatmal *et al*., the most commonly reported ADRs were tiredness (59.0%), followed by injection site pain and swelling (58.0%).^15^ These reactions had multiple risk factors, including age, gender, the health status of the participant, smoking status, type of COVID-19 vaccine and the number of doses. These 2 reactions were also the most common in this study; however, these 2 ADRs are very common in most vaccinations and are not specific to the COVID-19 vaccine.^16^

Recovery from the side effects caused by the different types of vaccines took 1–3 days in 48.0% of responders and 7.0% recovered on the same day. Among the respondents, 5.5% required medical attention after experiencing an ADR from the vaccines. Approximately 9.0% of participants required time off work for the day following the vaccination, and 11.6% reported to work but were still not feeling well. Although a high percentage did not feel well after vaccination, time taken as sick leave due to COVID-19 infection is much longer.

This study is the first to report such ADRs due to COVID-19 vaccines at SQUH and Oman at large. As with any other retrospective study, some limitations are inherent in this type of design. There were some missing questions in the survey sent to participants with one major question related to the brand of vaccine received by responders who did not experience an ADR. This affected the interpretation of the results and could be misleading if not properly interpreted. Additionally, the questionnaire sent was non-compulsory, hence the low rate of response by the participants. Those who did not experience any untoward side effects might not have been interested in completing any forms. Moreover, the study did not have a specific scale for severity and therefore severity was more subjective to symptoms and responders’ own opinion rather than an objective measurement.

## Conclusion

This observational retrospective study demonstrated the most common side effects experienced by both COVID-19 vaccines used at SQUH in Oman—Oxford-AstraZeneca and Pfizer-BioNTech. In this cohort, only mild symptoms were experienced, and females had more risk of ADRs compared to males. It is crucial to observe long-term ADRs to the vaccines and follow-up monitoring should be done to preclude any unwanted effects. Furthermore, spreading awareness of this type of vaccine is specifically recommended to enhance better uptake of the vaccine.

## References

[1] Polack FP, Thomas SJ, Kitchin N, Absalon J, Gurtman A, Lockhart S (2020). Safety and efficacy of the BNT162b2 mRNA Covid-19 vaccine. N Engl J Med.

[2] Borobia AM, Carcas AJ, Pérez-Olmeda M, Castaño L, Bertran MJ, García-Pérez J (2021). Immunogenicity and reactogenicity of BNT162b2 booster in ChAdOx1-S-primed participants (CombiVacS): A multicentre, open-label, randomised, controlled, phase 2 trial. Lancet.

[3] BioNTech A phase 3 study to evaluate the safety, tolerability, and immunogenicity of multiple production lots and dose levels of BNT162b2 RNA-based COVID-19 vaccines against COVID-19 in healthy participants.

[4] Ramasamy MN, Minassian AM, Ewer KJ, Flaxman AL, Folegatti PM, Owens DR (2021). Safety and immunogenicity of ChAdOx1 nCoV-19 vaccine administered in a prime-boost regimen in young and old adults (COV002): A single-blind, randomised, controlled, phase 2/3 trial. Lancet.

[5] Cristina M, Klaser K, May A, Polidori L, Capdevila J, Louca P (2021). Vaccine side-effects and SARS-CoV-2 infection after vaccination in users of the COVID symptom study app in the UK: A prospective observational study. Lancet Infect Dis.

[6] Kaur RJ, Dutta S, Bhardwaj P, Charan J, Dhingra S, Mitra P (2021). Adverse events reported from COVID-19 vaccine trials: A systematic review. Indian J Clin Biochem.

[7] Chapin-Bardales J, Gee J, Myers T (2021). Reactogenicity following receipt of mRNA-based COVID-19 vaccines. Jama.

[8] Tobaiqy AM, Elkout H, MacLure K (2021). Analysis of thrombotic adverse reactions of COVID-19 AstraZeneca vaccine reported to EudraVigilance database. Vaccines (Basel).

[9] Dutta S, Kaur RJ, Bhardwaj P, Sharma P, Ambwani S, Islam S (2021). Adverse events reported from the COVID-19 vaccines: A descriptive study based on the WHO database (VigiBase®). J Appl Pharm Sci.

[10] David SSB, Shamir-Stein N, Gez SB, Lerner U, Rahamim-Cohen D, Zohar AE (2021). Reactogenicity of a third BNT162b2 mRNA COVID-19 vaccine among immunocompromised individuals and seniors-A nationwide survey. Clin Immunol.

[11] Higashino T, Yamazaki Y, Senda S, Satou Y, Yonekura Y, Imai K (2022). Assessment of delayed large local reactions after the first dose of the SARS-CoV-2 mRNA-1273 vaccine in Japan. JAMA Dermatol.

[12] Klein NP, Lewis N, Goddard K, Fireman B, Zerbo O, Hanson KE (2021). Surveillance for adverse events after COVID-19 mRNA vaccination. JAMA.

[13] Pai M, Chan B, Stall NM, Grill A, Ivers N, Maltsev A (2021). Vaccine-induced immune thrombotic thrombocytopenia (VITT) following adenovirus vector COVID-19 vaccination. Sci Briefs Ontario COVID-19 Sci Advisory Table.

[14] Al-Maqbali JS, Al Rasbi S, Kashoub MS, Al Hinaai AM, Farhan H, Al Rawahi B (2022). A 59-year-old woman with extensive deep vein thrombosis and pulmonary thromboembolism 7 days following a first dose of the Pfizer-BioNTech BNT162b2 mRNA COVID-19 vaccine. American J Case Rep.

[15] Hatmal MM, Al-Hatamleh MA, Amin N, Olaimat RM, Fawaz M, Kateeb ET (2022). Reported adverse effects and attitudes among Arab populations following COVID-19 vaccination: a large-scale multinational study implementing machine learning tools in predicting post-vaccination adverse effects based on predisposing factors. Vaccines (Basel).

[16] Stone CA, Cosby A, Rukasin CRF, Beachkofsky TM, Phillips EJ (2019). Immune-mediated adverse reactions to vaccines. Br J Clin Pharmacol.

